# Binding of Perfluoroalkyl Substances to Nanoplastic Protein Corona Is pH‐Dependent and Attenuates Their Bioavailability and Toxicity

**DOI:** 10.1002/smsc.202400255

**Published:** 2024-09-23

**Authors:** Zongshan Zhao, Jiaqiang Yao, Haimei Li, Jing Lan, Yan Bao, Lining Zhao, Wansong Zong, Yanmin Long, Lei Feng, Henner Hollert, Xingchen Zhao

**Affiliations:** ^1^ College of Environmental Science and Engineering Qingdao University Qingdao 266071 China; ^2^ Qingdao Institute of Bioenergy and Bioprocess Technology Chinese Academy of Sciences Qingdao 266101 China; ^3^ College of Life Sciences Hebei University Baoding 071000 China; ^4^ College of Geography and Environment Shandong Normal University Jinan 250014 China; ^5^ Institute of Environment and Health Jianghan University Wuhan 430056 China; ^6^ School of Chemistry and Chemical Engineering Shandong University Jinan 250100 China; ^7^ Department for Evolutionary Ecology and Environmental Toxicology Goethe University Frankfurt am Main 60438 Germany; ^8^ Department of Environmental Media‐related Ecotoxicology Fraunhofer Institute for Molecular Biology and Applied Ecology Schmallenberg 57392 Germany; ^9^ Department for Evolutionary Ecology and Environmental Toxicology Goethe University Frankfurt am Main 60438 Germany

**Keywords:** cellular toxicity, nanoplastics, polyfluoroalkyl substances, protein, ternary interaction

## Abstract

There is a severe lack of understanding of the effects of micro/nanoplastics on human proteins and cells, especially in the presence of organic pollutants. Herein, both in vivo and in vitro assays are conducted to structurally evaluate blood protein complexed with perfluorooctanoic acid (PFOA) and perfluorooctane sulfonate (PFOS) as well as their coronas formed on polystyrene nanoplastics (PNs). PFOS is bound to serum albumin (SA) about 4 times as firmly as PFOA, which is not influenced by protein corona formation onto PN surfaces. However, the small molecular binding dramatically suppresses SA–PN aggregation. Low pH weakens the protein interaction of PFOS while not PFOA, which is also independent of PN adsorption, but the interaction with SA is still stronger for PFOS than PFOA, indicating higher serum persistence and risks. The presence of PN suppresses the cellular uptake of the chemicals and attenuates cytotoxicity due to low bioavailability. Overall, these results provide fundamental information on the ternary interaction mode of protein, particle, and organic pollutants in physiological environments with varying pH, as well as the subsequent cellular responses.

## Graphical abstract

This study explores how nanoplastics (NPs) influence the binding behavior of serum albumin (SA) in varying pH environments, noting that low pH reduces perfluorooctane sulfonate but not perfluorooctanoic acid interactions, independent of NP adsorption. Perfluoroalkyl and polyfluoroalkyl substances significantly inhibit SA–NP aggregation. Furthermore, PN presence reduces cellular uptake of chemicals, lessening cytotoxicity by lowering bioavailability.

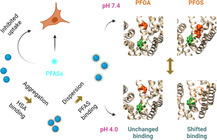

## 1 Introduction

Microplastics (MPs) with one dimension less than 5 mm have now been deemed an emerging pollutant and one of the defining indicators of the Anthropocene. In recent years, nanoplastics (NPs) have become the newest focus in the environmental arena after being found as intermediate substances before decomposing and degrading into oligomers and monomers. The high specific surface area of both plastic particles (PPs) increases the sorption of other contaminants with smaller dimensions from the surrounding environment, which makes them suitable as physical vectors for these pollutants through all the environmental matrixes, biota included.^[^ ^1^, ^2^ ^]^ Many studies have documented the ability of MPs and NPs as vectors for pollutants, especially for some hydrophilic ones, and these pollutant‐associated MPs have been found in real‐world samples.^[^ ^3^ ^]^

Biologically, MPs modify the overall bioavailability of the adsorbed pollutants, increasing or decreasing the toxicity of the latter, as a consequence of the possible different relations in the pollutant–plastic–organism ternary system. Per‐ and polyfluoroalkyl substances (PFASs) have high adsorption capacity on MPs under environmental conditions.^[^ ^4^ ^]^ Coexposure with NPs markedly increased the perfluorooctane sulfonate (PFOS) bioaccumulation and reactive oxygen species levels in mussel tissues, which led to antioxidative disorders and genetic alterations.^[^ ^5^ ^]^ Terrestrially, the coexistence of MPs significantly increased the bioaccumulation factor for perfluorooctanoic acid (PFOA) and PFOS by up to 200% in earthworms, remarkably reducing their reproduction.^[^ ^6^ ^]^ From the ecotoxicological point of view, the MP‐small molecular pollutant complex may show a change in toxicity concerning individual contaminants instead of a simple decrease or increase of toxicity.^[^ ^7^ ^]^ These phenomena suggest the need to go beyond the classical model and seek additive, synergistic, or antagonist effects.

There is a severe lack of understanding of their effects on human body components, although they are widely found in human tissues.^[^ ^8^, ^9^, ^10^ ^]^ Blood, considered a connective tissue, also serves as a vehicle or sink for PPs. There is a high possibility that these particles interact with the most abundant protein—serum albumin (SA).^[^ ^11^ ^]^ Thus, it is imperative to elucidate how PPs are associated with proteins and are transported through the human body. This study investigates the association between PFAS‐absorbed polystyrene nanoplastics (PNs) and human serum albumin (HSA) both in vivo and in vitro. The reciprocal influences of PFASs and PNs on the structure of SA were studied, and various characterization techniques were performed to disclose the interaction mechanisms among them. The binding kinetics of HSA to PFASs as a function of pH with or without PNs were also recorded. The impact mechanisms of either PFASs or PNs concentration on HSA stability were systematically investigated and discussed. The findings here provide fundamental insights into the interaction of the protein with the PFAS–PNs pollutant system. They are expected to enable a better understanding of the macromolecular impacts of pollutants in different biological fluids.

## 2 Results and Discussion

### 2.1 Identification of the Major Binding Protein and Its Interaction with PFASs

**Figure 1:**
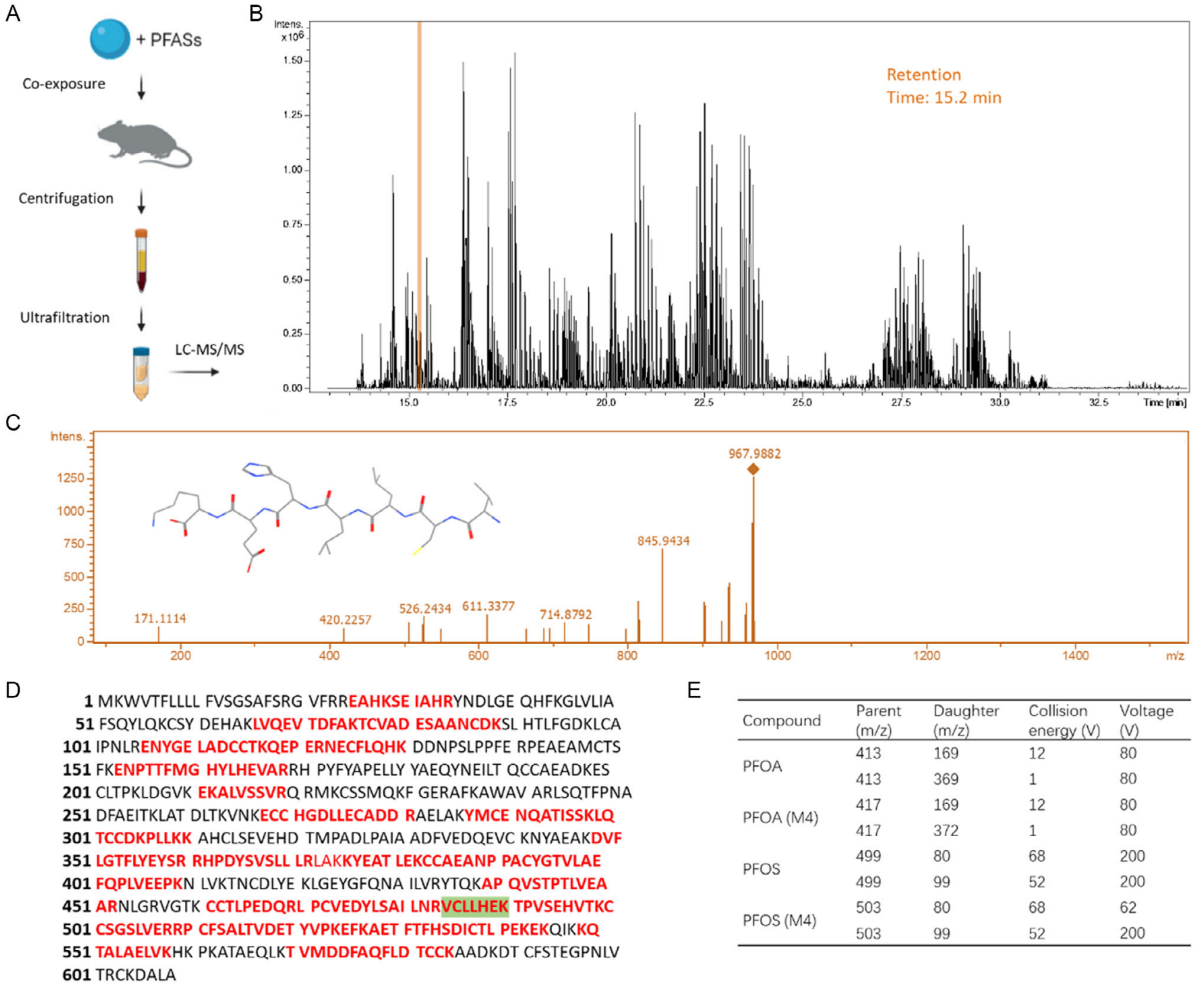
A) Scheme of the experimental procedure for isolating the protein and PFASs bound to PNs. B) LC spectrum of the trypsin‐digested protein collected from the filter residues. C) MS/MS data of the LC peak with a retention time of 15.2 min, which was characterized as the sequence of VCLLHEK (Val–Cys–Leu–Leu–His–Glu–Lys, highlighted with green background, with an inset showing the structure). D) The identified peptide sequences were shown and marked in red. E) Acquisition parameters for the detection of PFOA and PFOS.

We carried out in vivo exposure of both PFASs and PNs to identify the major binding protein. After injection of these pollutants in mice and balancing for half an hour, PNs in the plasma were separated and subjected to ultrafiltration. The filter residues were then subjected to liquid chromatography (LC)–mass spectrometry (MS)/MS and database research analysis (**Figure** **1**A). We found mouse SA as the major binding protein of PNs with the highest amino acid sequence coverage (Figure 1B–D).

Two pairs of ions were selected as quantification and confirmation transition ions for the presence of PFASs. The mass spectrum of PFOA revealed a molecular ion [*M*‐H]^−^ at 413 with major fragments of *m*/*z* 169 and 369 after analytes were infused into the mass spectrometer (Figure 1E). Data of PFOS revealed a deprotonated molecular ion [*M*‐H]^−^ at 499, along with major fragments of *m*/*z* 80 and 99. For PFOA (M4) and PFOS (M4), the mass transition of 417 to 372 and 503 to 80 was applied, respectively. All the data confirmed the binding of PFASs to the PN protein corona, which is aligned with previous reports. Han et al. found that over 90% of PFOA was bound to albumin in human or rat blood under physiological conditions using size exclusion chromatography and ligand binding techniques.^[^ ^12^ ^]^ Beesoon et al. confirmed higher binding affinities of PFOS and PFOA to total serum protein, of which HSA is known to be the major component.^[^ ^13^ ^]^ Therefore, in vitro analyses were further conducted to detail the interaction mode in simulated physiological environments.

### 2.2 Adsorption and Stability of the Protein Corona

**Figure 2:**
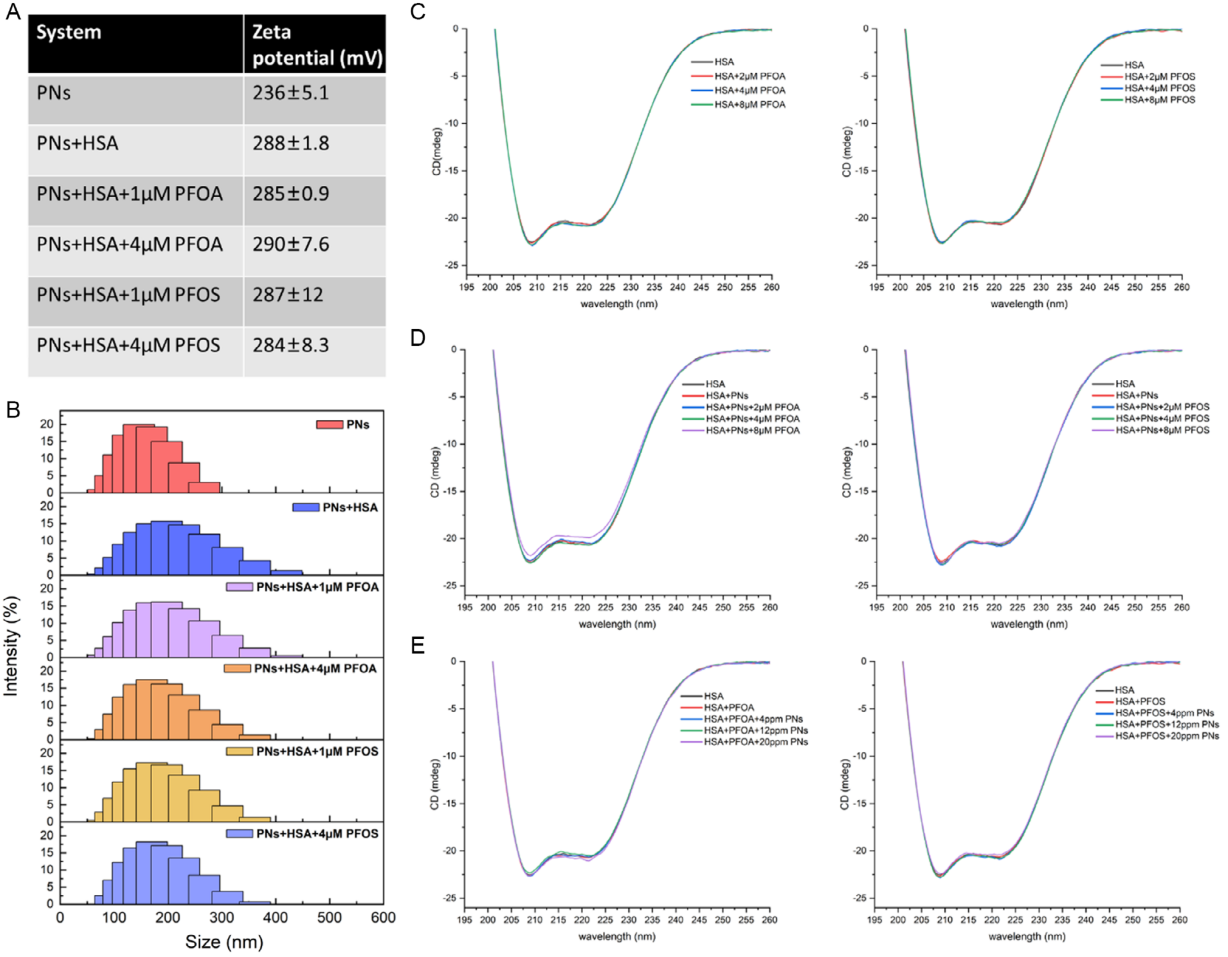
A) Zeta potential changes and B) DLS distribution of PNs in the absence or presence of HSA at different PFAS concentrations. PNs and HSA concentrations were kept at 1 μg mL^−1^ (0.374 pM) and 1 μm, respectively. C) CD spectra showing the structural changes of HSA following the addition of PFASs. HSA concentration was kept at 1 μm. D) CD spectra showing the structural changes of HSA following the addition of PFAS and PNs. PNs and HSA concentrations were kept at 1 μg mL^−1^ (0.374 pM) and 1 μm, respectively. E) CD spectra showing the structural changes of HSA following the addition of PFAS and PNs. PFAS and HSA concentrations were kept at 1 μm.

PNs are negatively charged with a diameter of 97.3 nm (Figure S1, Supporting Information). Dynamic light scattering (DLS) was used to monitor the size changes induced by self‐aggregation or protein adsorption with the influence of PFASs. The size change of PNs due to the addition of proteins is presented in **Figure** **2** and Figure S2A, Supporting Information. Changes following the addition of HSA were around 45 nm, which is significantly larger than the protein size, suggesting a probable thick protein layer and aggregation of PNs initiated by protein adsorption. The aggregation was getting gradually smaller with increasing concentration of PFASs, probably driven by PFAS dispersion and electrostatic repulsion. There seemed to be no differences between PFOA and PFOS. However, the circular dichroism (CD) spectrum observed for all the complexes showed almost no unfolding of the protein structure (Figure 2 and Figure S2B, Supporting Information). The distinction between HSA–PNs with or without introducing PFAS was not evidenced, showing local and adaptive structural changes occurred. Irrespective of the PN addition, the secondary structure of HSA in the presence of PFASs was unaffected. The surface charge of PNs increased after protein interaction and was almost unchanged with the addition of PFASs, attributed to the free energy minimization, which makes it stable and well suspended in blood.^[^ ^14^, ^15^ ^]^

### 2.3 Mode of Interaction

**Figure 3:**
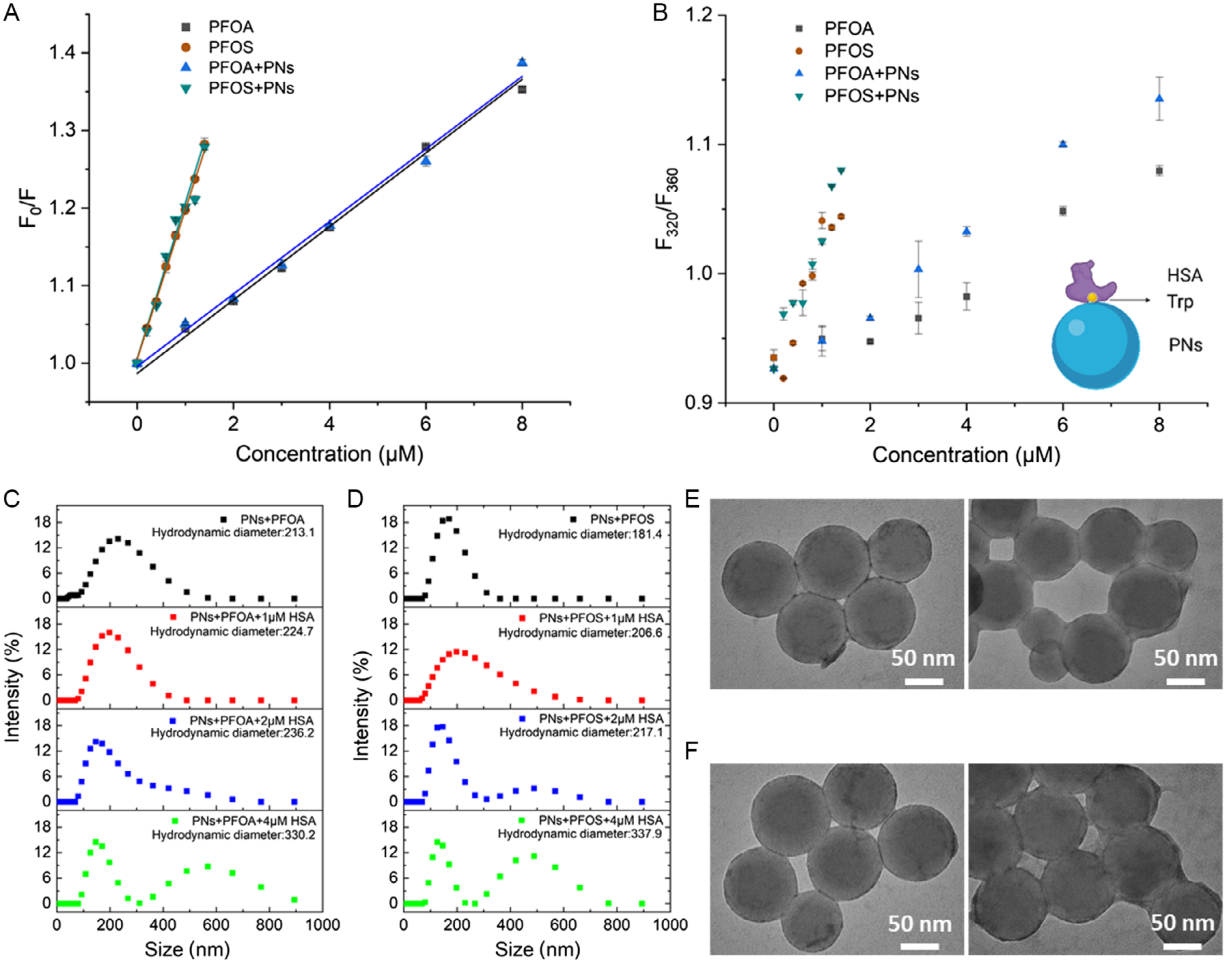
A) Stern–Volmer plots for HSA in the presence of increasing concentrations of PFASs. The concentration of PNs was set to be at 3 μg mL^−1^ (1.12 pM). B) The shift of Trp emission and proposed “side‐on” binding geometries. The inset illustration displays the binding mode. Data points are reported as mean values and error bars denote standard deviation of three runs. C,D) The hydrodynamic diameter distribution of PNs in the presence of PFASs with the addition of different amounts of HSA. The bimodal distribution indicates the bridging effects of HSA and easy formation of aggregates. PN and PFAS concentrations were kept at 3 μg mL^−1^ (1.12 pM) and 2 μm, respectively. The upper and lower images in E,F) are the transmission electron microscopy (TEM) images corresponding to the black and green samples in (C) and (D), respectively.

Fluorescence quenching revealed the possible binding modes by quantifying the quenching of fluorophores (Trp, Phe, and Tyr) by interacting with PN and PFASs. The fluorescence quenching efficiency depends on the relative accessibility of pollutants to the chromophore groups.^[^ ^16^, ^17^ ^]^ Here, we chose Trp as the sensor for the ligand binding. By selectively exciting Trp without influence on other fluorophores, we are able to obtain information on the binding behavior of the PFASs. A progressive decrease in the fluorescence intensity was observed due to the quenching of HSA fluorescence, indicating changes in the microenvironment of Trp‐214 upon pollutant binding. The linear curves were obtained by extracting the data and fitting it with the Stern–Volmer model (**Figure** **3**A and Figure S3, Supporting Information), which gives more quantitative information on the quenching capability. The obtained quenching constant (*K*_SV_) showed the presence of a dynamic quenching mode (Förster resonance energy transfer excluded, Figure S4, Supporting Information) but the different quenching ability of the PFAS types with or without PNs. The *K*_SV_ of PFOA‐associated interaction was 0.467 (with PNs) and 0.474 (without PNs) × 10^5^ M^−1^, respectively, while it was 2.005 (with PNs) and 1.926 (without PNs) × 10^5^ M^−1^ for PFOS, indicating a more substantial binding ability of the latter chemical (**Table** **1**). These measurements are consistent with the findings of Beesoon et al. who reported that the dissociation constant of PFOA (*K*_d_ = 1(±0.9) × 10^−4^ M) was much higher than that of PFOS (*K*_d_ = 8(±4) × 10^−8^ M), demonstrating a much weaker binding affinity of PFOA to HSA than PFOS.^[^ ^13^ ^]^

**Table 1:** Fitting parameters of the Stern–Volmer model for PFAS binding with HSA in the absence or presence of PNs.

	PFOA		PFOA + PNs		PFOS		PFOS + PNs	
pH	7.4	4.0	7.4	4.0	7.4	4.0	7.4	4.0
*K*_SV_ (×10^5^ M^−1^)	0.474	0.460	0.467	0.444	1.926	1.349	2.005	1.651
*K*_q_ (×10^13^ M^−1^ s^−1^)	0.948	0.920	0.934	0.888	3.852	2.698	4.010	3.302
*R* ^2a^	0.9958	0.9976	0.9915	0.9989	0.9995	0.9837	0.9529	0.9761

Low binding affinity increases the concentration of free PFOA in the blood, enhances the redistribution in the body, and enables faster pollutant excretion. On the contrary, high binding slows the release of PFOS from the “reservoir”—HSA, and increases the dwell time in organisms. Many endogenous substances in human blood can bind to HSA, such as bilirubin, fatty acids, vitamins, hormones, and electrolytes.^[^ ^18^ ^]^ They compete with pollutants for binding sites, and this competition can be completely ignored when the affinity of a pollutant exceeds a certain range.^[^ ^19^, ^20^ ^]^ The regulation of endogenous substances on pollutants–HSA interaction is mainly governed by the affinity between them. The greater the affinity, the smaller the impact of endogenous substances.^[^ ^21^, ^22^ ^]^ Thus, many strongly binding pollutants and drugs can replace the endogenous toxins, resulting in toxicity. For example, sulfonamide displaces bilirubin from the HSA binding sites, leading to an elevation of plasma bilirubin. The released bilirubin crosses the blood–brain barrier, reaches central neurons, and causes kernicterus.^[^ ^23^ ^]^

#### 2.3.1 R is the Correlation Coefficient

The spectral shift was further evaluated using the double wavelength method, where fluorescence intensity ratios at 320 and 360 nm (F320/F360) were plotted as a function of the PFAS concentration (Figure 3B). The ratio increased gradually and showed a positive relationship with the small molecule concentrations, suggesting a blueshift of the emission peak that can be considered as increased Trp hydrophobicity or stronger binding. There is an elevated F320/F360 value in the presence of PNs, indicating more changes in the Trp hydrophobicity following adsorption compared with small molecules alone. This phenomenon is in line with the synchronous fluorescence spectra, which document the information in the vicinity of Trp (Δ*λ* = 60). A decrease of the Trp intensity, together with a blue shift, was observed, indicating decreased polarity and nonpolar exposure around Trp‐214 (Figure S5, Supporting Information).

PNs did not substantially interact with Trp through intermolecular forces or energy transfer as PFASs did, as evidenced by negligible fluorescence quenching with increasing concentrations of PNs (Figure S6, Supporting Information). Given this information, HSA seemed to attach its triangular face and adopt a “side‐on” conformation on PNs, which created a hydrophobic environment for Trp located at the center of the protein.^[^ ^24^ ^]^ If more protein were added into the system by fixing PNs and PFASs, larger aggregates (Figure 3C–F) rather than well‐dispersed complex particles would be witnessed. In this case, PFASs were insufficient for HSA binding, and the dispersion effect became negligible.

**Figure 4:**
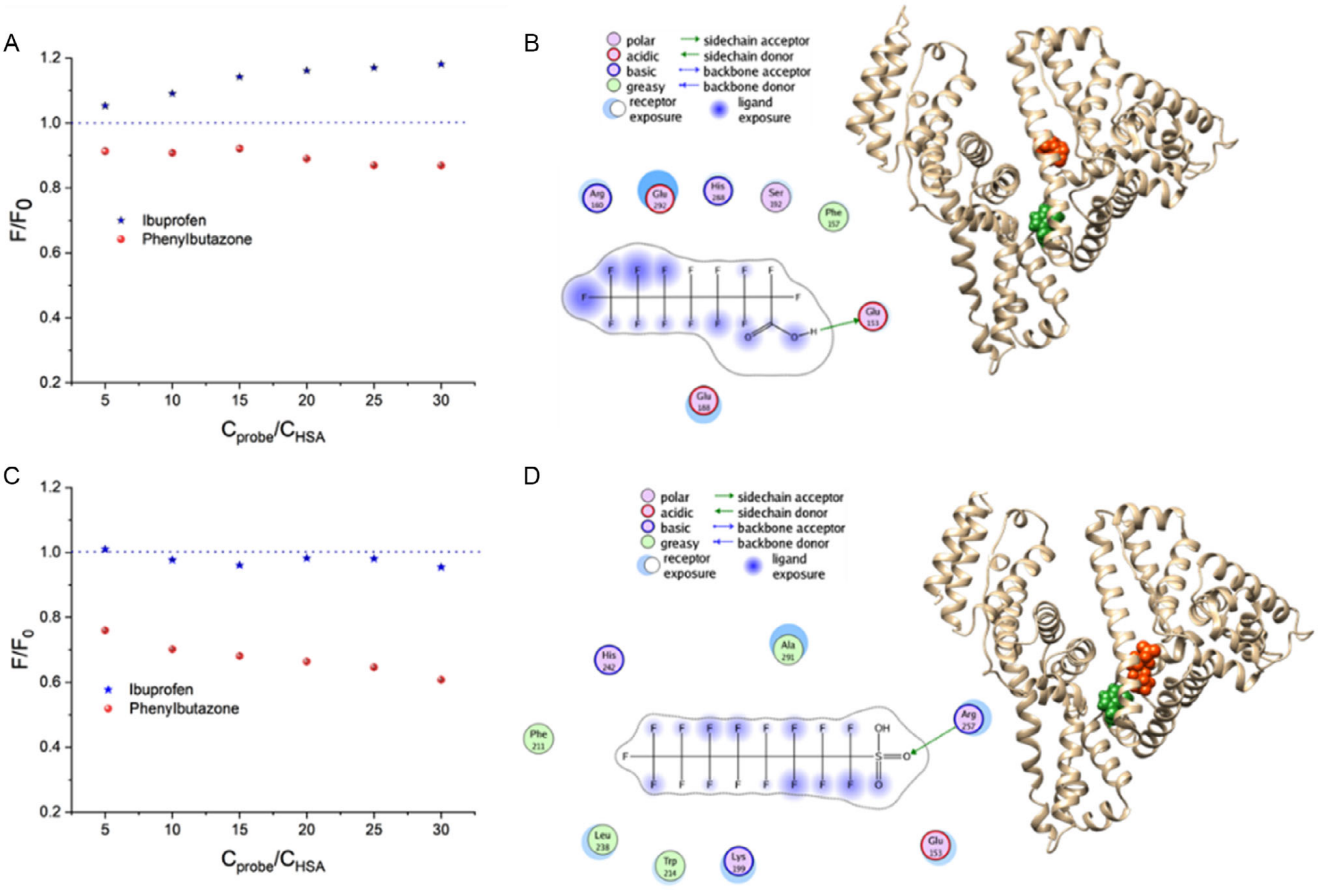
HSA titrated with probe molecules in the presence of PFOA A) and PFOS C). Concentrations of HSA, PFOA, and PFOS were 1, 2, and 20 μm. B,D) The molecular docking results of PFOA and PFOS with HSA, respectively. Trp was shown as the green residue in the center of the protein, and PFASs were shown as the orange molecule.

To find out the interaction mechanism and the accessibility of the interacting PFASs to the intrinsic fluorophore, site probe tests were carried out using phenylbutazone and ibuprofen, which specifically bind sites I and II, respectively. As displayed in **Figure** **4**A,C, the fluorescence of HSA–PFOS was remarkably quenched by phenylbutazone. It is assumed that PFOS is located in the subdomain IIA of HSA and was near Trp‐214, while PFOA was in neither site but in the vicinity. This was proved by molecular docking results of the prior binding site (Figure 4B,D and Table S1, S2, Supporting Information), where the shorter distance explained the higher quenching efficiency of PFOS (4.23 Å) than PFOA (12.85 Å). PFASs may also bind to the protein surfaces, as evidenced by the docking results (Figure S7, Supporting Information) with fewer scores, which may serve the dispersion effect as elucidated in the DLS study. However, there is no further fluorescence quenching in the presence of PNs. This phenomenon is different from our results of brominated flame retardants (BFRs), where adsorption of HSA onto PNs further quenched the intrinsic fluorescence of HSA–BFRs.^[^ ^25^ ^]^

### 2.4 pH‐Dependency of the Interaction

**Figure 5:**
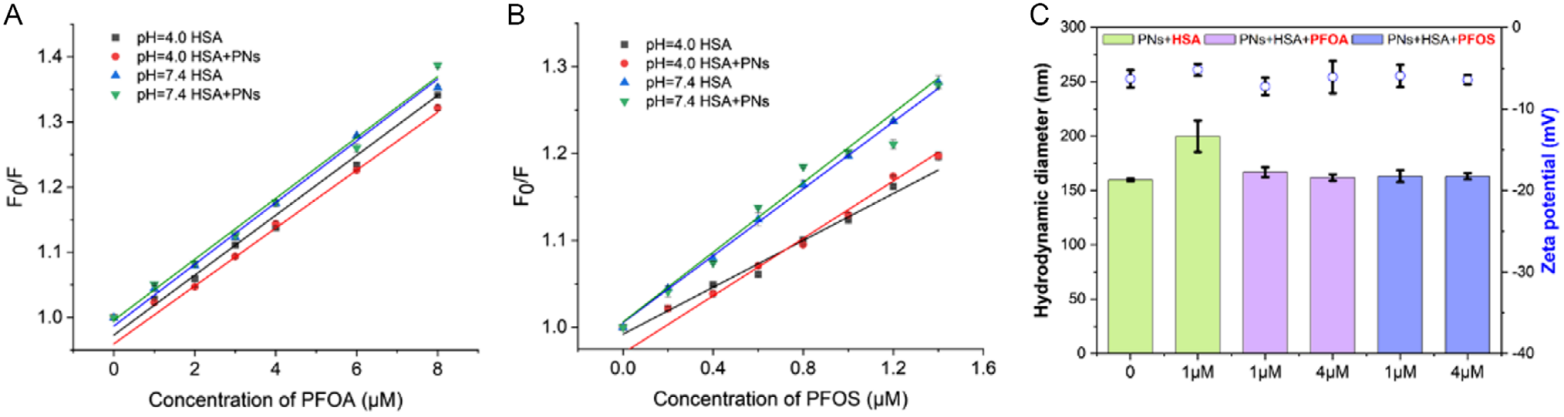
A) Stern–Volmer plots of HSA quenched by PFOA in the absence and presence of PNs at pH 7.4 and 4.0. B) Stern–Volmer plots of HSA quenched by PFOS in the absence and presence of PNs at pH 7.4 and 4.0. C) Hydrodynamic diameter and zeta potential changes of PNs due to the adsorption of HSA and PFAS interaction at pH 4.0. Data points are reported as mean values and error bars denote standard deviation from three runs. PNs and HSA concentrations were kept at 3 μg mL^−1^ (1.12 pM) and 1 μm, respectively, with varying amounts of PFASs added. Error bars represent the standard deviations of three experiments.

The interaction at pH 4.0 was also investigated, which is the pH in intestine environments.^[^ ^26^ ^]^ pH changes have almost no influence on the HSA–PFOA interaction in the presence or absence of PNs. However, at pH 4.0, the quenching by PFOS was markedly weaker, as documented by a decreased slope of the Stern–Volmer plots compared with pH 7.4 (**Figure** **5**A,B and Table 1). Still, PNs rarely impacted the protein–ligand interactions, the same with pH 7.4. HSA has a molecular weight of 67 kDa and an isoelectric point of 4.7.^[^ ^27^ ^]^Accordingly, HSA is positively charged at a pH 4.0. The unchanged fluorescence indicates the unaffected primary binding of PFOA at pH 4.0.^[^ ^28^, ^29^ ^]^ Comparably, the binding sites are assumed to shift dramatically due to the strong proton environment. Both PFOA and PFOS are good H‐bonding synthons because they can act as proton donors with —OH and can be acceptors with =O.^[^ ^30^ ^]^ Compared with PFOA, PFOS with sulfonic acid groups can form stronger hydrogen bonds as proton acceptors due to higher electronegativity. However, the strong proton environment at pH 4.0 weakened the deprotonation and H‐bonding formation of PFOS to a larger extent. The deformation of H‐bonding and rearrangement of electron cloud greatly influence the binding and fluorescence quenching.^[^ ^31^ ^]^ Besides, the enhanced acidity or electronegativity of PFOS compared to PFOA results primarily from the presence of the additional oxygen atom which provides a greater negative inductive effect to enhance ionization, which makes the interaction more sensitive to pH.

When HSA was subjected to the PN solution, there was an increment in hydrodynamic diameter (Figure 5C and Figure S8, Supporting Information). The positive charges on HSA may neutralize a large number of negative charges on PNs, and some of them may serve as a bridge to attach other PNs (i.e., bridging flocculation), as the diameter difference (about 45 nm) is much larger than the dimension of HSA (80 × 80 × 30 Å).^[^ ^32^ ^]^ The aggregates were dispersed by PFAS addition as was evidenced by decreased hydrodynamic diameter. This suggests that the addition of the PFAS induced dispersion, somehow interfering with the HSA bridging by electrostatic repulsive forces.

### 2.5 The Influence of PNs on PFAS Uptake and Cellular Toxicity

**Figure 6:**
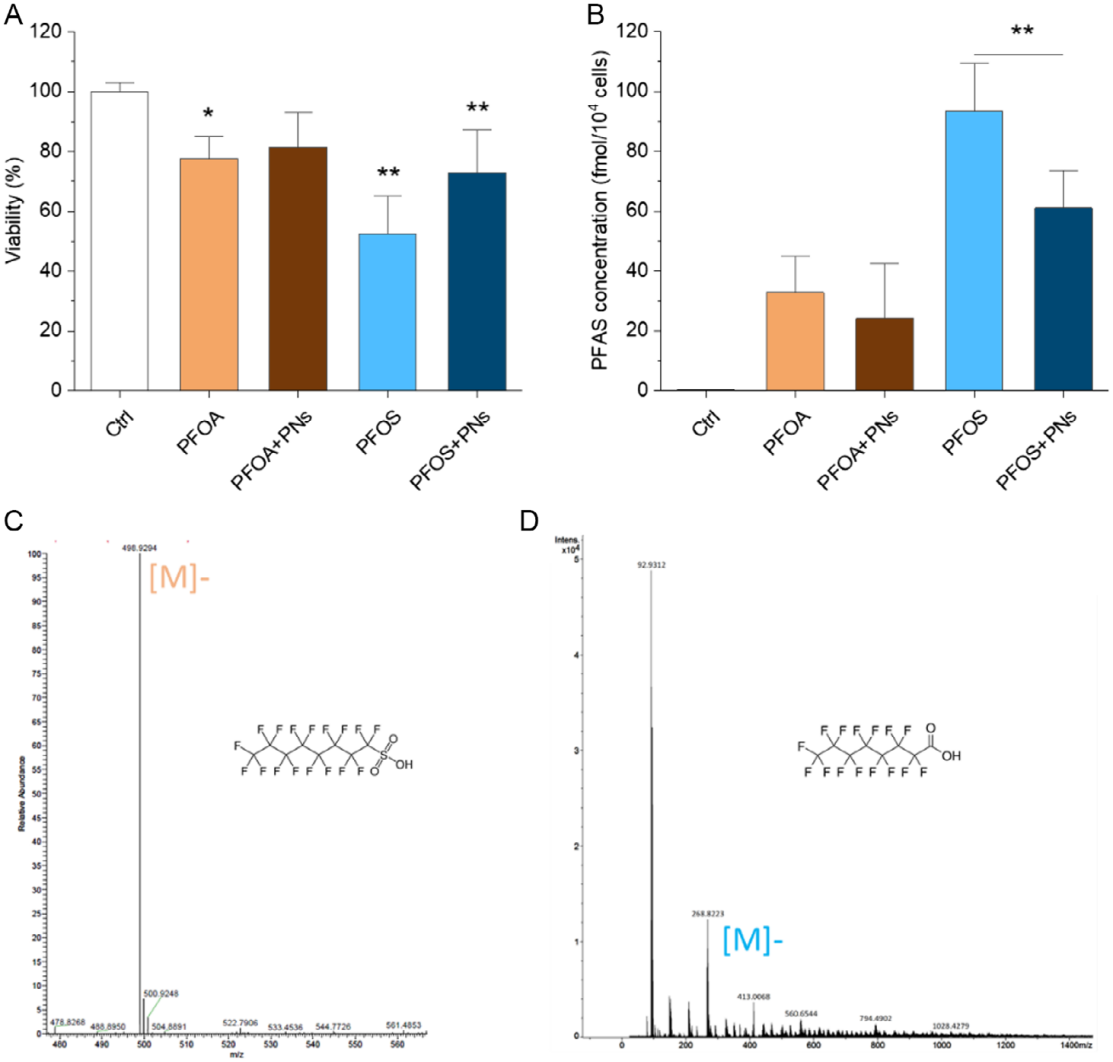
A) Relative viability of human lung epithelial A549 cells. Significance was shown between the control and treatment groups. B) PFAS concentrations in the cells. Data points are reported as mean values and error bars denote standard deviation from three independent runs. Statistical significance was evaluated using Tukey's post‐hoc analysis with significance expressed as *p* < 0.05* and *p* < 0.01**. The concentrations of PNs and PFASs were 50 μg mL^−1^ (18.7 pM) and 500 μm, respectively. Data were expressed as the mean ± SD. C,D) MS/MS spectra of PFOA and PFOS fragment patterns detected from PFAS‐exposed cells with parent ions shown as [M].

The joint exposure of PFASs with PNs was evaluated in quest of the biological sequences at a cellular level. Such interaction is also mediated with SA, which is used to supplement cell culture media and buffers the toxicity induced by PFASs because of protein binding.^[^ ^33^ ^]^ We chose human lung epithelial cells as the model because inhalation is essential to human exposure to both PFASs and MPs.^[^ ^34^, ^35^, ^36^ ^]^ Statistically significant differences were found between the exposure to PFASs and the blank control (**Figure** **6**A). Overall, there was little evidence of potentiation, synergy, or antagonistic effects from PNs on PFOA toxicity. Surprisingly, cytotoxicity endpoints showed considerably reduced PFOS toxicity for the combination with PNs (*p* = 0.030), even though there was essentially no difference for PFOA following the addition of PNs (*p* = 0.987). Recent research on the sorption of PFASs onto MPs found that electrostatic and hydrophobic interactions were the primary driving forces.^[^ ^37^ ^]^ Given the hydrophobic nature of perfluorocarbon moieties in PFASs and their high specific surface area, the adsorption onto the PNs could occur before interacting with the cells. In the culture media, the concentration of SA is around the magnitude of 10^−4^ M; thus, the stoichiometry of the pollutants and SA is comparable to that in the above ternary system. As was indicated, large aggregates tend to form at a low PN:SA ratio, making less cellular uptake of PNs due to the increased particle size. As a result, the interaction of the ternary system minimizes the potential uptake of the small molecules due to the comparable difficulty of PN uptake. The adsorption reduced the amount of free PFASs available to the cells, thus reducing the bioavailability of PFASs (Figure 6B). Nevertheless, the hydrophilicity of PFOA is greater than that of PFOS, and PFOA is expected to adsorb less and is also less influenced by PNs.

There is a very limited amount of scientific literature addressing the combined exposure of small molecular pollutants with micro/nanoplastics. As an example, the study of Soto‐Bielicka et al. and Shi et al. documented that the cytotoxicity of TBBPA and phthalate esters declined when coexposed with PNs.^[^ ^38^, ^39^ ^]^ On the other hand, a recent study using Caco‐2 cells reported higher toxicity when PFOS was coexposed with PNs, probably due to combined toxicity.^[^ ^40^ ^]^ The disparities in our results would be due to the different sizes of the PNs employed (20 versus 100 nm). The PNs used in their study already showed high toxicity. Moderate amounts of PNs can modify the behavior of PFASs and help to achieve high cellular viability. However, at a tissue, organ, or organism level, such toxicity attenuation effects may not be applicable because of the potential risks of aggregates.

## 3 Conclusions and Perspective

Small molecule pollutants, micro/nanoplastic, and biomolecule interactions modify the pollutants’ and biomolecules’ physicochemical/physiological properties and subsequent interactions with cells and organisms. In order to better understand the complex interaction, the combined pollution effects on the protein structure, particularly the influence of PNs on the interaction of HSA and PFASs, were studied under biologically relevant conditions. Fluorescence and CD spectroscopy have diagnosed the formation of protein corona. The secondary structure of the protein did not change after interacting with any substance, although PFOA and PFOS show different accessibility to the chromophore groups of the protein. Based on the size distribution and fluorescence quenching studies, the interaction mode differed at pH 4.0, highlighting a subtle increase in aggregate formation at light PFAS concentrations. The presence of PNs attenuated the cellular uptake of PFASs, which is related to the adsorption of PFAS molecules. As a result, the decreased bioavailability weakened the cytotoxicity of PFASs. As the binding of PFASs to HSA alone or in the presence of PN will strongly depend on the concentration ratios of three constituents in the mixture, our results may not be applicable to other concentration ratio scenarios.

Considering the complexity of biological or environmental media, the corona formed on NP surfaces is often involved in multiple biomacromolecules (e.g., lipids and proteins) interfering with endogenous (e.g., amino acids and carbohydrates) or exogenous (e.g., drugs, pollutants) small molecules. All these substances contribute to an evolving corona composition and cellular response. Besides, in parallel to structural analyses, protein functionality and the importance of plastic surface identity in terms of chemical adsorption would be crucial to illustrate the biological relevance of protein corona, and further study is needed.

## 4 Experimental Section

4.1

4.1.1

##### Reagents

PNs with a diameter of 100 nm were bought from Tianjin BaseLine ChromTech Research Center, China. To calculate the molar concentration of particles, the formula *M* = *N*/*N*_A_ is utilized, where *N* represents the number concentration of nanoparticles (in PNs/L), and the denominator is the Avogadro constant (*N*_A_ = 6.02 × 10^23^). In our case, PNs with a density of 1.06 g mL^−1^ have a total mass concentration of 100 mg L^−1^ in the stock solution, a corresponding particle number concentration of 2.25 × 10^13^ PNs/L, and a particle molarity of 37.4 pM. Fatty‐acid‐free HSA with more than 96% purity was purchased from Beijing Biotopped Co. Ltd., China. PFOA and PFOS were purchased from Mecklin Co., Ltd. (Shanghai, China) and LGC Standards Ltd. (Teddington, UK), respectively. Phenylbutazone (purity > 98%) and ibuprofen (purity > 98%) were all purchased from Shanghai Rhawn Chemical Technology Co., Ltd., China. Phosphate‐buffered saline powder was obtained from Sigma (St. Louis, USA) to make the final concentration 0.1 mol L^−1^, pH 7.4. Ultrapure water with a resistance of 18.2 MΩ·cm was used throughout the experiments.

##### In vivo Tests

The exposure methods of pollutants to C57BL/6 mice (Vital River Laboratory Animal Technology Co. Ltd. China) were adopted from our previous study with a dose of 1 × 10^−3^ mol and 500 mg kg^−1^ (187 pmol kg^−1^) of PFASs and PNs, respectively.^[^ ^41^ ^]^ The collected plasma was ultrafiltered using the dialysis membrane (Spectra/Por Biotech CE MWCO 1 000 000, Fischer Scientific, Waltham, USA) to remove any unbound protein. The filter residue was washed 3 times with PBS and was analyzed by electrospray LC MS/MS using the MicrOTOF‐QII (Bruker Daltonics, Karlsruhe, Germany) equipped with a Prominence Nano 2D HPLC system (Shimadzu, Tokyo, Japan) as described in our previous work.^[^ ^41^ ^]^

##### In vitro Tests

PFASs with a concentration range of 0–8 μm were applied to each sample because serum levels of PFOA and PFOS can reach as high as several hundred or thousand ng mL^−1^, which is roughly equivalent to the magnitude of μm.^[^ ^42^ ^]^ PFASs with various concentrations were added to PN solutions with a constant concentration of 3 μg mL^−1^ (1.12 pM). The mixtures were then incubated for 1 h until HSA was added to make the final concentration of the protein at 1 μm and were allowed for an extra incubation for 0.5 h. Also, changes in the diameter of the PFAS–PN interaction system were also studied in the presence of various concentration of HSA (2, 4, and 8 μm), and PN and PFAS concentrations were kept at 3 μg mL^−1^ (1.12 pM) and 2 μm, respectively. The as‐prepared samples were vortexed and placed in square polystyrene cuvettes and capillary cells, respectively, and then subjected to zeta potential and size distribution measurements with a Malvern Nano ZS90 Zetasizer (Malvern, UK) with a He/Ne laser (633 nm).

The CD spectrum was taken on a Chirascan CD Spectrometer (Applied Photophysics Ltd., UK), constantly purging with 99.99% oxygen‐free nitrogen at a pressure of 4–6 bar. 3 μg mL^−1^ (1.12 pM) of PNs was incubated with PFASs (concentrations: 2, 4, and 8 μm) for 1 h prior to interaction with HSA (1 μm) for 0.5 h. The spectra of the as‐prepared samples were recorded from 190 to 250 nm with background subtraction. Three parallel scans were averaged for each spectrum, the secondary structure of which was analyze using the BeStSel program online at https://bestsel.elte.hu/.

Fluorescence measurements were carried out on an F‐4500 Spectrofluorometer (Hitachi, Tokyo, Japan). The final concentrations of PFOA were 0, 1, 2, 3, 4, 6, and 8 μm and that of PFOS were 0, 0.2, 0.4, 0.6, 0.8, 1, 1.2, and 1.4 μm. To avoid the inner filter effect, the concentrations of PNs and HSA were set to be at 3 μg mL^−1^ (1.12 pM) and 1 μm, respectively. The excitation wavelength was selectively set at 295 nm (excitation wavelength for Trp only), and the emission spectra were recorded from 290 to 450 nm for steady‐state fluorescence spectra. The Stern–Volmer model was used to analyze the fluorescence intensity data.^[^ ^43^, ^44^, ^45^ ^]^

(1) $$\frac{F_{0}}{F}=1+K_{\text{SV}}\left[Q\right]=1+k_{q}\tau_{0}\left[Q\right]$$

where *F*_0_ and *F* are the fluorescence intensities in the absence and presence of pollutants, respectively; *k*_q_ is the quenching constant; *τ*_0_ is the lifetime of HSA with pollutants, and is about 5 ns.^[^ ^41^ ^]^ *K*_SV_ is the Stern–Volmer fluorescence quenching constant, reflecting the binding or quenching efficiency, and [*Q*] is the PFAS concentration. Synchronous fluorescence spectra were recorded at a wavelength interval of 60 nm.

The UV–vis absorption spectra of the solution containing both HSA and PFASs or PNs were measured using a UV‐1780 absorption spectrometer (Shimadzu, Tokyo, Japan) in the wavelength range of 200–400 nm in 1.0 cm quartz cells using a 1 nm slit width. The solutions of PFASs or PNs were used as references. The final concentrations of PFOA were 0, 1, 2, 3, 4, 6, and 8 μm and that of PFOS were 0, 0.2, 0.4, 0.6, 0.8, 1, 1.2, and 1.4 μm. The final concentration of HSA was kept at 1 μm.

Molecular modeling of HSA and PFASs was conducted using MOE2008.10 (Chemical Computing Ltd., Montreal, Canada). The structure data of HSA (PDB ID:1BJ5, resolution: 2.50 Å) and PFOA/PFOS were obtained from the Protein Data Bank (rcsb.org) and PubChem (https://pubchem.ncbi.nlm.nih.gov/), respectively. The docking method was adopted from our previous work.^[^ ^46^ ^]^ The results were illustrated using UCSF Chimera1.16.^[^ ^47^ ^]^

For TEM analysis, PN (3 μg mL^−1^ or 1.12 pM) solutions with HSA (1 μm) and PFASs (2 μm) were incubated overnight, and the mixture or PN solution samples were prepared by being dropped onto 300 mesh formvar carbon‐coated copper grids (Electron Microscopy China, Beijing, China) and left overnight. The images were collected using the FEI Talos F200S TEM (Hillsboro, USA) at 200 keV.

Culturing of human lung epithelial A549 cells was maintained in DMEM media (Gibco, Waltham, USA) with 10% fetal bovine serum, 50 I.U. mL^−1^ penicillin and 50 μg mL^−1^ streptomycin (Gibco, Waltham, USA) in a humidified incubator at 37 °C and 5% CO_2_. Cells were sown in 96‐well plates with 100 mL of cell media and 1 × 10^4^ cells per well for exposure studies. A compound medium containing PFASs (500 μm) with or without PNs (50 μg mL^−1^ or 18.7 pM, preincubated with PFASs for 3 days) was substituted for the original culture medium after 12 h. A medium containing 0.1% DMSO was used as a vehicle control. After 48 h of incubation, cells were treated with trypsin, and the viability was determined using the trypan blue assay on a Thermo Fisher Countess II automated cell counter (Waltham, USA).

A previously established procedure was used to determine PFASs extracted from cells.^[^ ^33^ ^]^ The cell pellets were washed twice with PBS after the samples were centrifuged at 200 g for 4 min. To extract PFASs from the cell pellets, 1 mL of acetonitrile was added and vortexed for 30 min. After centrifuging the supernatant at 12 000 g for 10 min, a 0.9 mL aliquot was obtained. The samples were kept at −20 °C until an HPLC–MS/MS analysis was performed in the negative mode. A standard working curve was used to calculate the amount of PFASs present in cells.

##### Statistical Analysis

Data were presented as mean ± SD with a sample size of 6 for each statistical analysis. Multiple comparisons were performed using a two‐way ANOVA and Tukey's post‐hoc analysis with significance against the control expressed as *p* < 0.05* and *p* < 0.01**. Data were analyzed and plotted using Origin software (Northampton, USA; version 2021b).

## Conflict of Interest

The authors declare no conflict of interest.

## Author Contributions

**Zongshan Zhao**: Data curation (lead). **Jiaqiang Yao**: Data curation (equal). **Haimei Li**: Formal analysis (lead). **Jing Lan**: Formal analysis (supporting). **Yan Bao**: Methodology (supporting). **Lining Zhao**: Funding acquisition (supporting). **Wansong Zong**: Methodology (supporting). **Yanmin Long**: Data curation (supporting). **Lei Feng**: Methodology (equal). **Henner Hollert**: Validation (lead). **Xingchen Zhao**: Conceptualization (lead); Investigation (lead); Writing—original draft (lead).

## Supporting information

Supplementary Material

## Data Availability

The data that support the findings of this study are available from the corresponding author upon reasonable request.
