# Supplementary material for: Binding of Perfluoroalkyl Substances to Nanoplastic Protein Corona Is pH‐Dependent and Attenuates Their Bioavailability and Toxicity
Source: Small Sci. 2024 Sep 23;4(12):2400255. doi: 10.1002/smsc.202400255 (PMC11935290; doi:10.1002/smsc.202400255)
Supplement: Supplementary file 1 — Supplementary Material [file SMSC-4-2400255-s001.pdf]

Supporting Information

**Binding of perfluoroalkyl substances to nanoplastic protein corona is pH-dependent and attenuates their bioavailability and toxicity**

*Zongshan Zhao, Jiaqiang Yao, Haimei Li, Jing Lan, Yan Bao, Lining Zhao, WansongZong, Yanmin Long, Lei Feng, HennerHollert, and Xingchen Zhao**

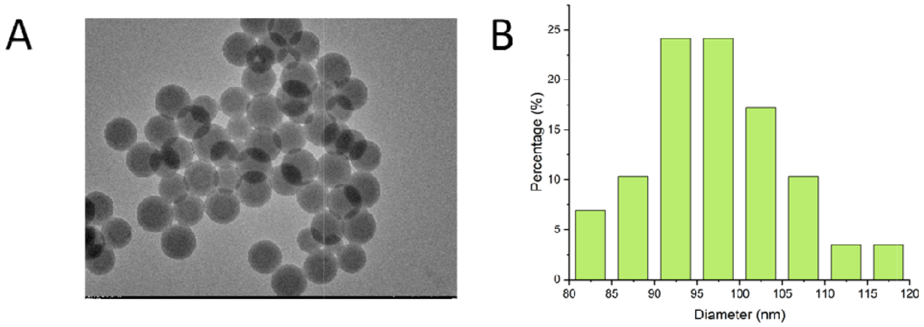


Figure S1.(A) TEM images and (B) distribution of the raw PNs.

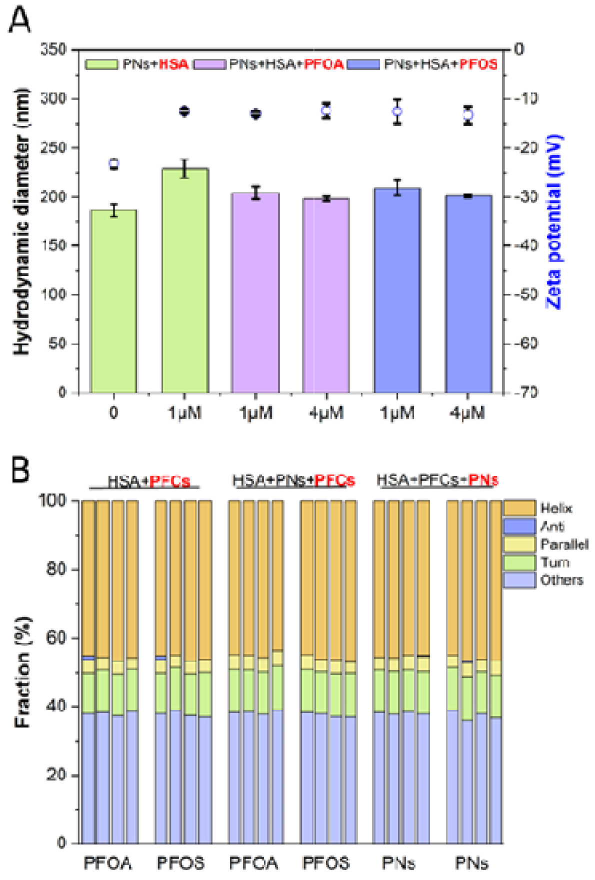


Figure S2.(A) Hydrodynamic diameter and zeta potential changes of PNs due to the adsorption of HSA and PFAS interaction at pH 7. Green bars: PNs with HSA (0 and 1 µM); purple bars: PNs with HSA (1 µM) and PFOA (1 and 4 µM); blue bars: PNs with HSA (1 µM) and PFOS (1 and 4 µM).PNs concentration was kept at 3 µg/mL(1.12 pM). Data points arereported as mean values and error bars denote standard deviation of three experiments. (B) Secondary structural contents of HSA with varying amounts of PFASs (left to right: 0, 2, 4, and 8 µM), HSA with PNs (12 µg/mL or 4.48 pM), and varying amounts of PFASs (left to right: 0, 2, 4, and 8 µM), HSA with PFASs (8 µM) and varying amounts of PNs (left to right: 0, 4, 12, and 20 µg/mL; or 0, 1.50, 4.48, and 7.48 pM).

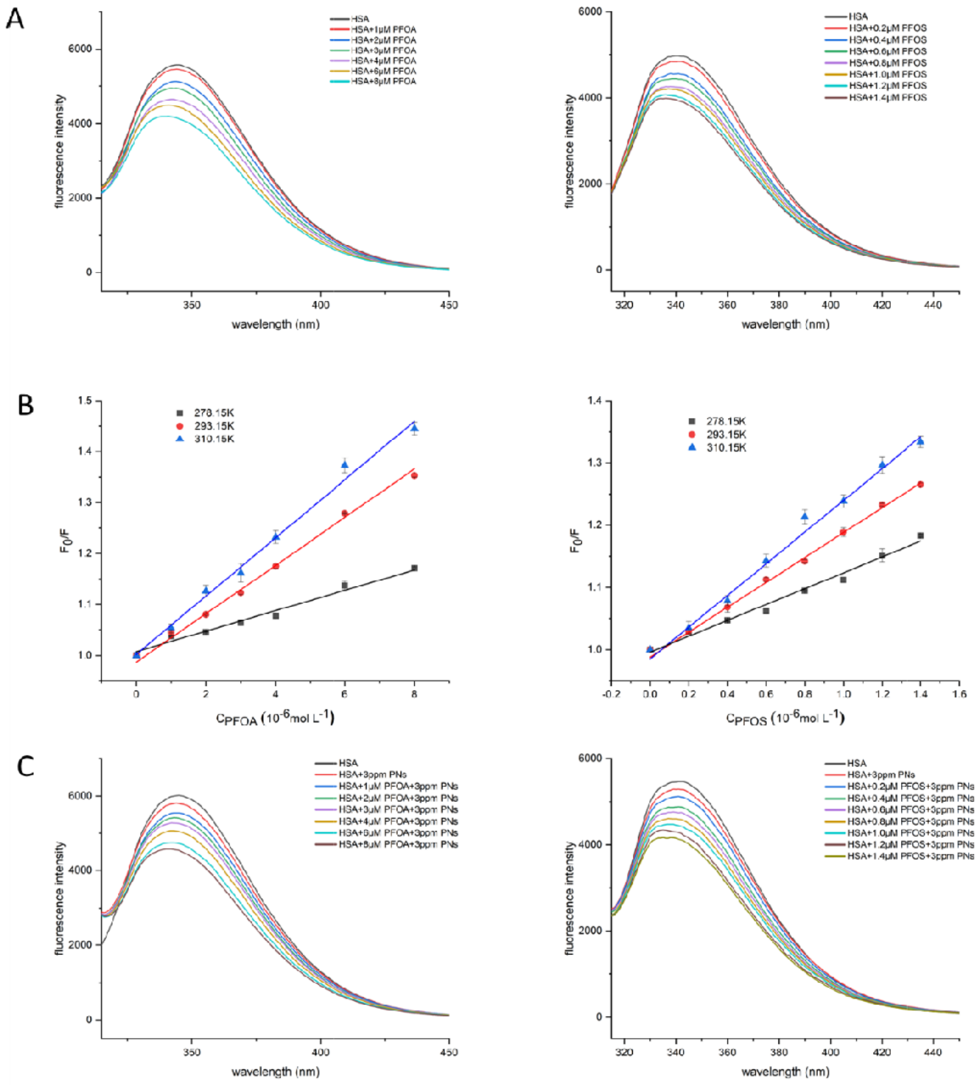


Figure S3.(A) Trp fluorescence emission of HSA (1 μM) in the presence of varyingPFAS concentration. (B) Stern-Volmer plots at different temperatures. (C) HSA (1 μM) fluorescence influenced by PNs (3 μg/mLor 1.12 pM) and varyingPFAS concentration.

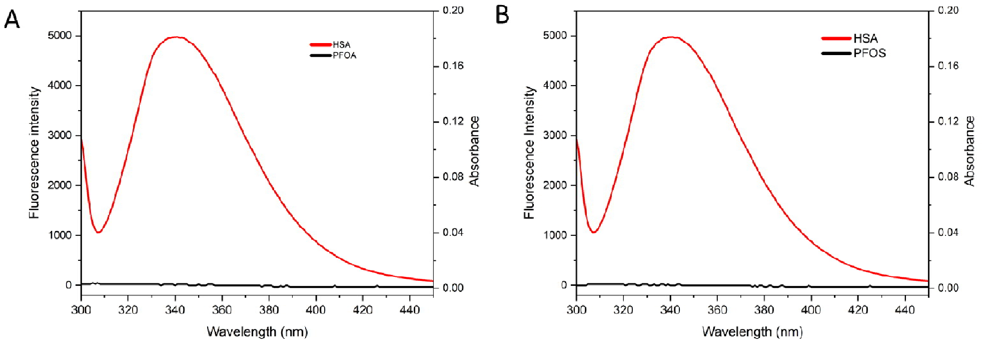


Figure S4.The overlaps of PFOA (A) and PFOS (B) absorption spectra and HSA fluorescence spectrum show limited FRET possibility.

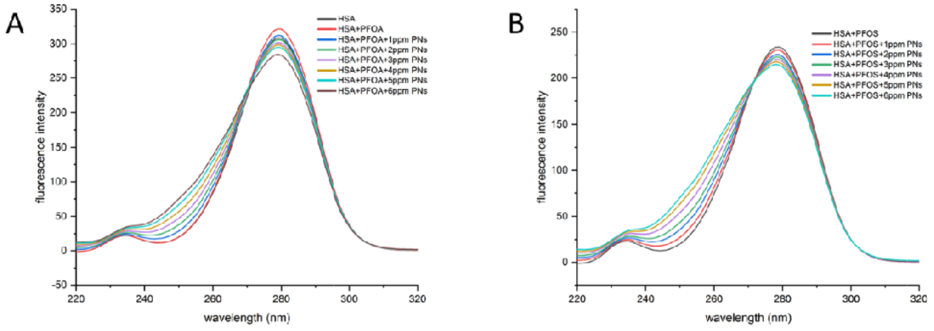


Figure S5.Synchronous fluorescence spectra of HSA + PFOA (A) or PFOS(B) in the presence of PNs when Δλ = 60. $c_{HSA} = c_{PFASs} = 1$ μM.

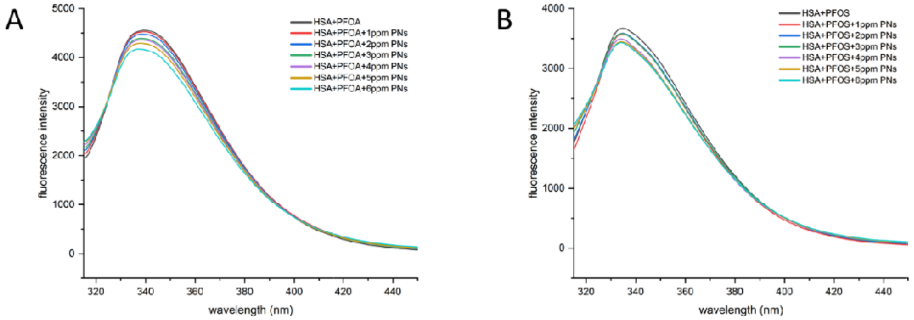


Figure S6. Trp fluorescence emission of HSA+ PFOA (A) or PFOS (B) in the presence of varyingPN concentration. $c_{HSA} = c_{PFASs} = 1$ μM.

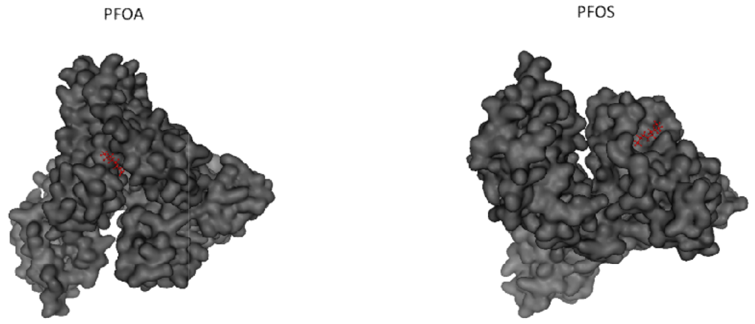


Figure S7.Exemplary docking results showing possible binding sites of PFOA (left) and PFOS (right) on the surface of the protein molecule.

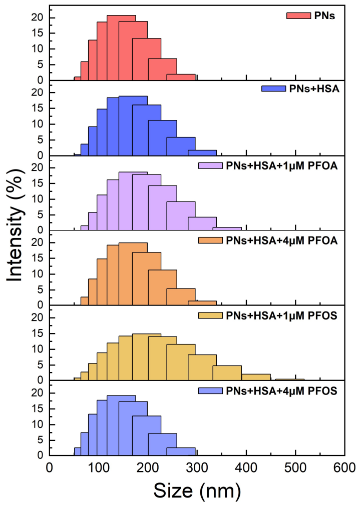


Figure S8. DLS distribution of PNs in the absence or presence of HSA at different PFAS concentrations. PNs and HSA concentrations were kept at 1 μg/mL(0.374 pM) and 1 μM, respectively. pH=4.

Table S1. Molecular docking parameters of HSA-PFOA interaction.

| ligand | receptor | residue | chain | type | score | distance |
|---|---|---|---|---|---|---|
| H 9222 | OE 2413 | GLU 153 | 4l8u 1 | H-don | 35.40% | 1.77 |
| F 9207 | OE 2413 | GLU 153 | 4l8u 1 | weak | 0.00% | 3.9 |
| F 9208 | OE 2413 | GLU 153 | 4l8u 1 | weak | 0.00% | 2.45 |
| F 9208 | OE 2412 | GLU 153 | 4l8u 1 | weak | 0.00% | 4.15 |
| F 9208 | CD 2411 | GLU 153 | 4l8u 1 | weak | 0.00% | 3.12 |
| F 9208 | CG 2408 | GLU 153 | 4l8u 1 | weak | 0.00% | 3.36 |
| O 9212 | OE 2412 | GLU 153 | 4l8u 1 | weak | 0.00% | 3.96 |
| O 9212 | CD 2411 | GLU 153 | 4l8u 1 | weak | 0.00% | 3.57 |
| C 9219 | OE 2413 | GLU 153 | 4l8u 1 | weak | 0.00% | 3.44 |
| C 9219 | CD 2411 | GLU 153 | 4l8u 1 | weak | 0.00% | 4.31 |
| C 9221 | OE 2413 | GLU 153 | 4l8u 1 | weak | 0.00% | 3.56 |
| C 9221 | CD 2411 | GLU 153 | 4l8u 1 | weak | 0.00% | 4.48 |
| F 9207 | CZ 2490 | PHE 157 | 4l8u 1 | weak | 0.00% | 3.23 |
| F 9207 | CE 2488 | PHE 157 | 4l8u 1 | weak | 0.00% | 3.67 |
| F 9207 | CE 2486 | PHE 157 | 4l8u 1 | weak | 0.00% | 4.17 |
| F 9208 | CZ 2490 | PHE 157 | 4l8u 1 | weak | 0.00% | 3.82 |
| F 9208 | CE 2488 | PHE 157 | 4l8u 1 | weak | 0.00% | 3.44 |
| C 9219 | CZ 2490 | PHE 157 | 4l8u 1 | weak | 0.00% | 4.16 |
| C 9219 | CE 2488 | PHE 157 | 4l8u 1 | weak | 0.00% | 4.21 |

| | | | | | | | |
|---|---|---|---|---|---|---|---|
| F 9198 | NH 2545 | ARG 160 | 4l8u 1 | weak | 0.00% | | 2.9 |
| F 9198 | NH 2542 | ARG 160 | 4l8u 1 | weak | 0.00% | | 4.5 |
| F 9198 | CZ 2541 | ARG 160 | 4l8u 1 | weak | 0.00% | | 3.98 |
| F 9206 | NH 2545 | ARG 160 | 4l8u 1 | weak | 0.00% | | 3.23 |
| F 9206 | NH 2542 | ARG 160 | 4l8u 1 | weak | 0.00% | | 3.57 |
| F 9206 | CZ 2541 | ARG 160 | 4l8u 1 | weak | 0.00% | | 3.45 |
| F 9206 | NE 2539 | ARG 160 | 4l8u 1 | weak | 0.00% | | 4.23 |
| C 9214 | NH 2545 | ARG 160 | 4l8u 1 | weak | 0.00% | | 4.15 |
| C 9218 | NH 2545 | ARG 160 | 4l8u 1 | weak | 0.00% | | 4.39 |
| F 9197 | OE 2970 | GLU 188 | 4l8u 1 | weak | 0.00% | | 4.29 |
| F 9197 | OE 2969 | GLU 188 | 4l8u 1 | weak | 0.00% | | 2.25 |
| F 9197 | CD 2968 | GLU 188 | 4l8u 1 | weak | 0.00% | | 3.29 |
| F 9197 | CG 2965 | GLU 188 | 4l8u 1 | weak | 0.00% | | 3.84 |
| F 9197 | CB 2962 | GLU 188 | 4l8u 1 | weak | 0.00% | | 4.07 |
| F 9198 | OE 2970 | GLU 188 | 4l8u 1 | weak | 0.00% | | 4.22 |
| F 9198 | OE 2969 | GLU 188 | 4l8u 1 | weak | 0.00% | | 2.43 |
| F 9198 | CD 2968 | GLU 188 | 4l8u 1 | weak | 0.00% | | 3.63 |
| F 9201 | OE 2969 | GLU 188 | 4l8u 1 | weak | 0.00% | | 4.21 |
| F 9202 | OE 2970 | GLU 188 | 4l8u 1 | weak | 0.00% | | 3.27 |
| F 9202 | OE 2969 | GLU 188 | 4l8u 1 | weak | 0.00% | | 2.46 |
| F 9202 | CD 2968 | GLU 188 | 4l8u 1 | weak | 0.00% | | 2.92 |

| | | | | | | |
|---|---|---|---|---|---|---|
| F 9202 | CG 2965 | GLU 188 | 4l8u 1 | weak | 0.00% | 3.9 |
| F 9206 | OE 2970 | GLU 188 | 4l8u 1 | weak | 0.00% | 3.81 |
| F 9206 | OE 2969 | GLU 188 | 4l8u 1 | weak | 0.00% | 3.35 |
| F 9206 | CD 2968 | GLU 188 | 4l8u 1 | weak | 0.00% | 3.98 |
| F 9207 | OE 2969 | GLU 188 | 4l8u 1 | weak | 0.00% | 3.45 |
| F 9207 | CB 2962 | GLU 188 | 4l8u 1 | weak | 0.00% | 4.27 |
| C 9214 | OE 2969 | GLU 188 | 4l8u 1 | weak | 0.00% | 2.71 |
| C 9214 | CD 2968 | GLU 188 | 4l8u 1 | weak | 0.00% | 3.86 |
| C 9215 | OE 2969 | GLU 188 | 4l8u 1 | weak | 0.00% | 4.14 |
| C 9216 | OE 2970 | GLU 188 | 4l8u 1 | weak | 0.00% | 4.33 |
| C 9216 | OE 2969 | GLU 188 | 4l8u 1 | weak | 0.00% | 3.08 |
| C 9216 | CD 2968 | GLU 188 | 4l8u 1 | weak | 0.00% | 3.92 |
| C 9218 | OE 2969 | GLU 188 | 4l8u 1 | weak | 0.00% | 3.82 |
| O 9212 | OG 3019 | SER 192 | 4l8u 1 | weak | 0.00% | 3.25 |
| O 9212 | CB 3016 | SER 192 | 4l8u 1 | weak | 0.00% | 3.63 |
| C 9221 | OG 3019 | SER 192 | 4l8u 1 | weak | 0.00% | 4.16 |
| C 9221 | CB 3016 | SER 192 | 4l8u 1 | weak | 0.00% | 4.13 |
| F 9203 | NE 4531 | HIS 288 | 4l8u 1 | weak | 0.00% | 3.31 |
| F 9203 | CE 4529 | HIS 288 | 4l8u 1 | weak | 0.00% | 4.18 |
| F 9203 | CD 4526 | HIS 288 | 4l8u 1 | weak | 0.00% | 3.97 |
| F 9204 | NE 4531 | HIS 288 | 4l8u 1 | weak | 0.00% | 3.2 |

| | | | | | | | |
|---|---|---|---|---|---|---|---|
| F 9204 | CE 4529 | HIS 288 | 4l8u 1 | weak | 0.00% | 3.79 |
| F 9204 | CD 4526 | HIS 288 | 4l8u 1 | weak | 0.00% | 4.38 |
| F 9208 | NE 4531 | HIS 288 | 4l8u 1 | weak | 0.00% | 4.07 |
| C 9217 | NE 4531 | HIS 288 | 4l8u 1 | weak | 0.00% | 3.89 |
| F 9198 | OE 4586 | GLU 292 | 4l8u 1 | weak | 0.00% | 4.08 |
| F 9199 | OE 4586 | GLU 292 | 4l8u 1 | weak | 0.00% | 3.49 |
| F 9199 | CD 4584 | GLU 292 | 4l8u 1 | weak | 0.00% | 4.41 |
| F 9200 | OE 4586 | GLU 292 | 4l8u 1 | weak | 0.00% | 1.69 |
| F 9200 | OE 4585 | GLU 292 | 4l8u 1 | weak | 0.00% | 3.48 |
| F 9200 | CD 4584 | GLU 292 | 4l8u 1 | weak | 0.00% | 2.67 |
| F 9200 | CG 4581 | GLU 292 | 4l8u 1 | weak | 0.00% | 3.54 |
| F 9201 | OE 4586 | GLU 292 | 4l8u 1 | weak | 0.00% | 4.46 |
| F 9203 | OE 4586 | GLU 292 | 4l8u 1 | weak | 0.00% | 2.56 |
| F 9203 | CD 4584 | GLU 292 | 4l8u 1 | weak | 0.00% | 3.56 |
| F 9203 | CG 4581 | GLU 292 | 4l8u 1 | weak | 0.00% | 3.88 |
| F 9204 | OE 4586 | GLU 292 | 4l8u 1 | weak | 0.00% | 2.86 |
| F 9204 | CD 4584 | GLU 292 | 4l8u 1 | weak | 0.00% | 4.08 |
| F 9209 | OE 4586 | GLU 292 | 4l8u 1 | weak | 0.00% | 3.18 |
| F 9209 | OE 4585 | GLU 292 | 4l8u 1 | weak | 0.00% | 3.39 |
| F 9209 | CD 4584 | GLU 292 | 4l8u 1 | weak | 0.00% | 3.33 |
| F 9209 | CG 4581 | GLU 292 | 4l8u 1 | weak | 0.00% | 4.26 |

| | | | | | | | |
|---|---|---|---|---|---|---|---|
| F 9210 | OE 4586 | GLU | 292 | 4l8u 1 | weak | 0.00% | 2.61 |
| F 9210 | OE 4585 | GLU | 292 | 4l8u 1 | weak | 0.00% | 3.22 |
| F 9210 | CD 4584 | GLU | 292 | 4l8u 1 | weak | 0.00% | 3.24 |
| F 9211 | OE 4586 | GLU | 292 | 4l8u 1 | weak | 0.00% | 4.38 |
| F 9211 | OE 4585 | GLU | 292 | 4l8u 1 | weak | 0.00% | 4.07 |
| C 9214 | OE 4586 | GLU | 292 | 4l8u 1 | weak | 0.00% | 3.86 |
| C 9215 | OE 4586 | GLU | 292 | 4l8u 1 | weak | 0.00% | 2.75 |
| C 9215 | CD 4584 | GLU | 292 | 4l8u 1 | weak | 0.00% | 3.88 |
| C 9216 | OE 4586 | GLU | 292 | 4l8u 1 | weak | 0.00% | 4.4 |
| C 9217 | OE 4586 | GLU | 292 | 4l8u 1 | weak | 0.00% | 2.9 |
| C 9217 | CD 4584 | GLU | 292 | 4l8u 1 | weak | 0.00% | 4.12 |
| C 9218 | OE 4586 | GLU | 292 | 4l8u 1 | weak | 0.00% | 4.44 |
| C 9219 | OE 4586 | GLU | 292 | 4l8u 1 | weak | 0.00% | 4.41 |
| C 9220 | OE 4586 | GLU | 292 | 4l8u 1 | weak | 0.00% | 3.45 |
| C 9220 | OE 4585 | GLU | 292 | 4l8u 1 | weak | 0.00% | 3.79 |
| C 9220 | CD 4584 | GLU | 292 | 4l8u 1 | weak | 0.00% | 3.87 |

Table S2. Molecular docking parameters of HSA-PFOS interaction.

| ligand | receptor | residue | | chain | type | score | distance |
|---|---|---|---|---|---|---|---|
| O 9216 | NH 4045 | ARG | 257 | 4l8u 1 | H-acc | 34.30% | 2.92 |
| O 9216 | NH 4042 | ARG | 257 | 4l8u 1 | H-acc | 10.50% | 3.16 |

| | | | | | | |
|---|---|---|---|---|---|---|
| S 9197 | OE 2412 | GLU 153 | 4l8u 1 | weak | 0.00% | 3.52 |
| S 9197 | CD 2411 | GLU 153 | 4l8u 1 | weak | 0.00% | 4.06 |
| F 9209 | OE 2412 | GLU 153 | 4l8u 1 | weak | 0.00% | 3.7 |
| O 9216 | OE 2412 | GLU 153 | 4l8u 1 | weak | 0.00% | 3.36 |
| O 9216 | CD 2411 | GLU 153 | 4l8u 1 | weak | 0.00% | 4.05 |
| O 9216 | CG 2408 | GLU 153 | 4l8u 1 | weak | 0.00% | 4.34 |
| O 9216 | CB 2405 | GLU 153 | 4l8u 1 | weak | 0.00% | 4.24 |
| O 9217 | OE 2413 | GLU 153 | 4l8u 1 | weak | 0.00% | 3.63 |
| O 9217 | OE 2412 | GLU 153 | 4l8u 1 | weak | 0.00% | 3.22 |
| O 9217 | CD 2411 | GLU 153 | 4l8u 1 | weak | 0.00% | 3.37 |
| O 9217 | CG 2408 | GLU 153 | 4l8u 1 | weak | 0.00% | 4.06 |
| C 9223 | OE 2412 | GLU 153 | 4l8u 1 | weak | 0.00% | 4.26 |
| F 9205 | CE 3139 | LYS 199 | 4l8u 1 | weak | 0.00% | 4.22 |
| F 9205 | CD 3136 | LYS 199 | 4l8u 1 | weak | 0.00% | 4.41 |
| F 9206 | NZ 3142 | LYS 199 | 4l8u 1 | weak | 0.00% | 3.52 |
| F 9206 | CE 3139 | LYS 199 | 4l8u 1 | weak | 0.00% | 3.51 |
| F 9206 | CD 3136 | LYS 199 | 4l8u 1 | weak | 0.00% | 4.17 |
| F 9211 | NZ 3142 | LYS 199 | 4l8u 1 | weak | 0.00% | 2.53 |
| F 9211 | CE 3139 | LYS 199 | 4l8u 1 | weak | 0.00% | 3.35 |
| F 9213 | NZ 3142 | LYS 199 | 4l8u 1 | weak | 0.00% | 3.97 |
| F 9213 | CE 3139 | LYS 199 | 4l8u 1 | weak | 0.00% | 4.21 |

| | | | | | | | |
|---|---|---|---|---|---|---|---|
| F 9214 | NZ 3142 | LYS 199 | 4l8u 1 | weak | 0.00% | 3.5 |
| F 9214 | CE 3139 | LYS 199 | 4l8u 1 | weak | 0.00% | 3.06 |
| F 9214 | CD 3136 | LYS 199 | 4l8u 1 | weak | 0.00% | 4.35 |
| C 9222 | NZ 3142 | LYS 199 | 4l8u 1 | weak | 0.00% | 4.19 |
| C 9222 | CE 3139 | LYS 199 | 4l8u 1 | weak | 0.00% | 4.41 |
| C 9224 | NZ 3142 | LYS 199 | 4l8u 1 | weak | 0.00% | 3.68 |
| C 9224 | CE 3139 | LYS 199 | 4l8u 1 | weak | 0.00% | 4.11 |
| C 9225 | NZ 3142 | LYS 199 | 4l8u 1 | weak | 0.00% | 3.95 |
| C 9225 | CE 3139 | LYS 199 | 4l8u 1 | weak | 0.00% | 3.99 |
| F 9213 | CZ 3329 | PHE 211 | 4l8u 1 | weak | 0.00% | 3.69 |
| F 9207 | CH 3385 | TRP 214 | 4l8u 1 | weak | 0.00% | 4.11 |
| F 9210 | CH 3385 | TRP 214 | 4l8u 1 | weak | 0.00% | 4.23 |
| F 9210 | CZ 3383 | TRP 214 | 4l8u 1 | weak | 0.00% | 4.1 |
| F 9211 | CH 3385 | TRP 214 | 4l8u 1 | weak | 0.00% | 3.21 |
| F 9211 | CZ 3383 | TRP 214 | 4l8u 1 | weak | 0.00% | 3.27 |
| F 9211 | CZ 3381 | TRP 214 | 4l8u 1 | weak | 0.00% | 4.35 |
| F 9211 | CE 3377 | TRP 214 | 4l8u 1 | weak | 0.00% | 4.46 |
| C 9224 | CH 3385 | TRP 214 | 4l8u 1 | weak | 0.00% | 4.24 |
| C 9224 | CZ 3383 | TRP 214 | 4l8u 1 | weak | 0.00% | 4.31 |
| F 9203 | CD 3769 | LEU 238 | 4l8u 1 | weak | 0.00% | 4.35 |
| F 9210 | CD 3769 | LEU 238 | 4l8u 1 | weak | 0.00% | 3.26 |

| | | | | | | |
|---|---|---|---|---|---|---|
| F 9210 | CD 3765 | LEU 238 | 4l8u 1 | weak | 0.00% | 3.25 |
| F 9210 | CG 3763 | LEU 238 | 4l8u 1 | weak | 0.00% | 3.72 |
| F 9211 | CD 3765 | LEU 238 | 4l8u 1 | weak | 0.00% | 4.49 |
| F 9212 | CD 3769 | LEU 238 | 4l8u 1 | weak | 0.00% | 3.2 |
| F 9213 | CD 3769 | LEU 238 | 4l8u 1 | weak | 0.00% | 3.12 |
| F 9213 | CD 3765 | LEU 238 | 4l8u 1 | weak | 0.00% | 3.91 |
| F 9213 | CG 3763 | LEU 238 | 4l8u 1 | weak | 0.00% | 4.11 |
| F 9213 | O 3759 | LEU 238 | 4l8u 1 | weak | 0.00% | 4.33 |
| C 9224 | CD 3769 | LEU 238 | 4l8u 1 | weak | 0.00% | 4.03 |
| C 9224 | CD 3765 | LEU 238 | 4l8u 1 | weak | 0.00% | 4.32 |
| C 9225 | CD 3769 | LEU 238 | 4l8u 1 | weak | 0.00% | 3.6 |
| F 9213 | ND 3837 | HIS 242 | 4l8u 1 | weak | 0.00% | 4.05 |
| F 9213 | CG 3834 | HIS 242 | 4l8u 1 | weak | 0.00% | 3.94 |
| F 9213 | CB 3831 | HIS 242 | 4l8u 1 | weak | 0.00% | 3.58 |
| S 9197 | NH 4045 | ARG 257 | 4l8u 1 | weak | 0.00% | 3.84 |
| S 9197 | NH 4042 | ARG 257 | 4l8u 1 | weak | 0.00% | 4.07 |
| S 9197 | CZ 4041 | ARG 257 | 4l8u 1 | weak | 0.00% | 4.44 |
| F 9199 | NH 4045 | ARG 257 | 4l8u 1 | weak | 0.00% | 4.21 |
| F 9199 | NH 4042 | ARG 257 | 4l8u 1 | weak | 0.00% | 4.29 |
| F 9202 | NH 4045 | ARG 257 | 4l8u 1 | weak | 0.00% | 4.45 |
| F 9208 | NH 4045 | ARG 257 | 4l8u 1 | weak | 0.00% | 2.87 |

| | | | | | | | |
|---|---|---|---|---|---|---|---|
| F 9208 | NH 4042 | ARG 257 | 4l8u 1 | weak | 0.00% | 4.21 |
| F 9208 | CZ 4041 | ARG 257 | 4l8u 1 | weak | 0.00% | 3.93 |
| F 9209 | NH 4045 | ARG 257 | 4l8u 1 | weak | 0.00% | 4.2 |
| O 9215 | NH 4045 | ARG 257 | 4l8u 1 | weak | 0.00% | 4 |
| O 9215 | NH 4042 | ARG 257 | 4l8u 1 | weak | 0.00% | 3.61 |
| O 9215 | CZ 4041 | ARG 257 | 4l8u 1 | weak | 0.00% | 4.27 |
| O 9216 | CZ 4041 | ARG 257 | 4l8u 1 | weak | 0.00% | 3.43 |
| C 9223 | NH 4045 | ARG 257 | 4l8u 1 | weak | 0.00% | 3.82 |
| F 9198 | CB 4568 | ALA 291 | 4l8u 1 | weak | 0.00% | 2.71 |
| F 9198 | O 4567 | ALA 291 | 4l8u 1 | weak | 0.00% | 3.67 |
| F 9198 | C 4566 | ALA 291 | 4l8u 1 | weak | 0.00% | 3.94 |
| F 9198 | CA 4564 | ALA 291 | 4l8u 1 | weak | 0.00% | 3.51 |
| F 9199 | CB 4568 | ALA 291 | 4l8u 1 | weak | 0.00% | 2.85 |
| F 9199 | CA 4564 | ALA 291 | 4l8u 1 | weak | 0.00% | 4.22 |
| F 9201 | CB 4568 | ALA 291 | 4l8u 1 | weak | 0.00% | 3.62 |
| F 9203 | CB 4568 | ALA 291 | 4l8u 1 | weak | 0.00% | 4.2 |
| O 9215 | CB 4568 | ALA 291 | 4l8u 1 | weak | 0.00% | 3.57 |
| C 9218 | CB 4568 | ALA 291 | 4l8u 1 | weak | 0.00% | 3.36 |
| C 9219 | CB 4568 | ALA 291 | 4l8u 1 | weak | 0.00% | 4.13 |
| C 9220 | CB 4568 | ALA 291 | 4l8u 1 | weak | 0.00% | 4.44 |
